# Increased risk of essential tremor in migraine: A population-based retrospective cohort study

**DOI:** 10.1371/journal.pone.0173586

**Published:** 2017-03-13

**Authors:** Chi-Ieong Lau, Che-Chen Lin, Hsuan-Ju Chen, Han-Cheng Wang, Wei-Hung Chen, Ji-An Liang

**Affiliations:** 1 Department of Neurology, Shin Kong Wu Ho-Su Memorial Hospital, Taipei, Taiwan; 2 Division of Clinical Neurology, Nuffield Department of Clinical Neurosciences, University of Oxford, Oxford, United Kingdom; 3 Institute of Cognitive Neuroscience, University College London, Queen Square, London, United Kingdom; 4 College of Medicine, Fu-Jen Catholic University, Taipei, Taiwan; 5 Management Office for Health Data, China Medical University Hospital, Taichung, Taiwan; 6 Healthcare Service Research Center (HSRC), Taichung Veterans General Hospital, Taichung, Taiwan; 7 College of Medicine, China Medical University, Taichung, Taiwan; 8 College of Medicine, Taipei Medical University, Taipei, Taiwan; 9 College of Medicine, National Taiwan University, Taipei, Taiwan; 10 Graduate Institute of Clinical Medical Science, School of Medicine, College of Medicine, China Medical University, Taichung, Taiwan; 11 Department of Radiation Oncology, China Medical University Hospital, Taichung, Taiwan; Icahn School of Medicine at Mount Sinai, UNITED STATES

## Abstract

**Purpose:**

To examine the long-term risk of essential tremor (ET) in migraine.

**Methods:**

Using population-based administrative data from a subset of the National Health Insurance Research Database (NHIRD) of Taiwan, we identified 22,696 newly diagnosed migraineurs (mean age 44.5 years) and a matched migraine-free cohort of 90,784 individuals in the period 2000–2008. Multivariable Cox proportional hazards regression analysis was conducted for assessing the ET risk for the migraine cohort compared to the migraine-free cohort.

**Results:**

After adjusting for covariates, the migraine cohort had a 1.83-fold increased risk (95% CI 1.50–2.23) of subsequent ET in comparison to the migraine-free cohort (8.97 vs. 4.81 per 10,000 person-years). In the subgroup analysis, patients with migraine were associated with higher risks of ET, regardless of gender, age or the existence of comorbidities.

**Conclusion:**

Our findings demonstrated an association between migraine and ET, suggesting a possible shared pathophysiology underpinning both disorders.

## Introduction

Both migraine and essential tremor (ET) are common neurological disorders. Over the past two decades, there has been controversy regarding the possible link between the two disorders [1–6]. In an early cross-sectional study, Biary et al. disclosed a bi-directional association between migraine and ET, with more than one-third of ET patients coexisted with migraine and almost one-fifth of migraineurs had ET [1]. Another study also showed the co-segregation of classical migraine in one-fourth of patients with hereditary essential tremor [2]. Together both studies suggest comorbidity between migraine and ET, yet a common criticism argued that they suffered from suboptimal comparisons with uncontrolled patient features such as age and sex [6]. More recently, however, two age- and sex-matched case-control studies reported conflicting results [4, 5]. A Chinese study disclosed a higher prevalence of lifetime migraine (22%) among 150 patients with ET compared with 150 controls (12.7%) [5]. By contrast, Barbanti et al. revealed no significant difference in the frequency of lifetime migraine between 110 patients with ET and 110 matched controls [4]. Hu et al. concluded that the contradictory results might be due to the different ethnic background of the studied populations as well as the differences in patient characteristics [5].

To date, there has been little agreement on the potential link between migraine and ET, with no large population-based studies existing in the literature. Exploring their relationship may shed light on the incompletely understood pathophysiology of both disorders as well as their possible shared mechanism. To this end, we conducted a nation-wide population-based cohort study from the NHIRD of Taiwan, to address the question of whether migraine is associated with subsequent ET.

## Methods

### Data source

The National Health Research Institutes (NHRI) built the NHIRD which involved the reimbursement claims data from the National Health Insurance (NHI) program of Taiwan. The Taiwan NHI was established by the Government and performed as a single-payer and compulsory health insurance that covers 99% of the 23 million population in Taiwan. The Longitudinal Health Insurance Database (LHID) is a subset of the NHRID constructed from one million randomly sampled insured individuals. According to the NHRI, there was no significant difference between the LHID and the NHIRD in respect to age and sex distribution. The LHID consists of annual claims data including the beneficiary registries, prescriptions as well as all outpatient and inpatient records. Disease records are based on the International Classification of Diseases, Ninth Revision, Clinical Modification (ICD-9-CM). All data and related metadata were deposited in an appropriate public repository in the National Health Research Institutes (NHRI). The data on the study population that were obtained from the NHIRD (http://nhird.nhri.org.tw/en/index.html) are maintained in the NHIRD (http://nhird.nhri.org.tw/). The NHRI is a nonprofit foundation established by the government. Only citizens of the Republic of China who fulfill the requirements of conducting research projects are eligible to apply for the NHIRD. The use of NHIRD is limited to research purposes only. Applicants must follow the Computer-Processed Personal Data Protection Law (http://www.winklerpartners.com/?p=987) and related regulations of National Health Insurance Administration and NHRI, and an agreement must be signed by the applicant and his/her supervisor upon application submission. All applications are reviewed for approval of data release.

### Ethics statement

Before releasing the LHID for the purpose of research, the NHRI eliminated the original identification information and encrypted an anonymous number for each individual in order to ensure privacy. The NHIRD encrypts patient personal information to protect privacy and provides researchers with anonymous identification numbers associated with relevant claims information, including sex, date of birth, medical services received, and prescriptions. Therefore, patient consent is not required to access the NHIRD. This study was approved to fulfill the condition for exemption by the Institutional Review Board (IRB) of China Medical University (CMUH104-REC2-115-CR1). The IRB also specifically waived the consent requirement.

### Study population

We conducted a population-based cohort study designed to explore the association between ET and migraine. The migraine cohort consisted of all newly diagnosed migraine patients aged ≥ 20 years old (ICD-9-CM 346) from January 1, 2000 to December 31, 2008. The initial diagnosis date was assigned as the index date of the migraine cohort. We identified the migraine-free cohort by propensity score (PS) matching [7]. Logistic regression model was used to estimate the probability of migraine assignment by calculating the PS. We incorporated age, sex, hypertension, diabetes mellitus (DM), coronary artery disease (CAD), stroke, epilepsy, head injury, restless leg syndrome (RLS), depression, dementia, asthma, chronic obstructive pulmonary disease (COPD), and year of index date in the logistic regression model. For each migraine case, the 4-fold corresponding migraine-free controls were selected from the nearest propensity score by greedy algorithm. We excluded the individuals with the history of ET diagnosis (ICD-9-CM 333.1) before the index date as well as individuals with Parkinson’s disease (ICD-9-CM 332) before the end of follow-up. Each individual was observed in both cohorts beginning at the index date until a diagnosis of ET (ICD-9-CM 333.1) was made, a subject being withdrawn from the Taiwan NHI or the end of the follow-up (December 31, 2011).

We considered the following comorbidities as the confounding factors of the current study. A comorbidity of an individual is defined as having the comorbidity before the index date. These included hypertension (ICD-9-CM 401–405), DM (ICD-9-CM 250), CAD (ICD-9-CM 410–414), stroke (ICD-9-CM 430–438), epilepsy (ICD-9-CM 345), head injury (ICD-9-CM 310.2, 800, 801, 803, 804, 850–854, and 959.01), RLS (ICD-9-CM 333.90, and 333.99), depression (ICD-9-CM 296.2–296.3, 300.4 and 311), dementia (ICD-9-CM 290, 294.1 and 331.0), asthma (ICD-9-CM 493), and. COPD (ICD-9-CM 491, 492, and 496). In addition, migraine prophylaxis used by the individuals during the period of follow-up (including amitriptyline, clomipramine, duloxetine, flunarizine, fluoxetine, gabapentin, imipramine, paroxetine, sertraline, topiramate, valproate, venlafaxine and beta blockers) were also considered as confounding factors.

### Statistical analysis

Data were expressed as frequencies and percentages for categorical data and as means and standard deviations for continuous variables. We compared the distributions of age, sex, and comorbidity between the migraine cohort and the migraine-free cohort. A value of 0.1 standardized mean difference or less indicates a negligible difference in means between two cohorts by using standardized mean difference [8]. We calculated the incidence density of ET for each study cohort and measured the cumulative incidence curves by using the Kaplan-Meier method. To test the incidence curve difference between the migraine and the migraine-free cohorts, we applied the log-rank test. To compare the risk for developing ET between the migraine and the migraine-free cohorts, we used single variable and multi-variable Cox proportional hazard models to estimate the crude and adjusted hazard ratios (HRs) and the corresponding 95% confidence intervals (CIs). We also performed a subgroup analysis between the migraine and the migraine-free cohorts as stratified by age, sex, and comorbidities. We applied SAS 9.4 software (SAS Institute, Cary, NC, USA) for data management and used R software (R Foundation for Statistical computing, Vienna, Austria) for statistical analysis and incidence curve calculation. We set the p-value at less than 0.05 as the significant level for two-side testing.

## Results

This study involved a total of 22,696 migraine subjects and a 4-fold migraine-free cohort (N = 90,784) (Table 1). The mean age of the study population was 44.5 years and the majority of cases were female (72%). There were no significant differences between the migraine cohort and migraine-free cohort in the distributions of age, sex and the presence of comorbidities. The migraine cohort had a higher proportion of use of migraine prophylaxis than the migraine-free cohort.

**Table 1:** Baseline demographic status and comorbidity compared between comparison and migraine cohorts.

Variable	Migraine-free cohort N = 90784 (%)	Migraine cohort N = 22696 (%)	Standardized mean difference
**Age, years (SD)**	44.5 (16.4)	44.5 (14.9)	0.002
**Sex**			0.029
Female	66862 (73.6)	16422 (72.4)	
Male	23922 (26.4)	6274 (27.6)	
**Comorbidity**			
Hypertension	23335 (25.7)	6013 (26.5)	0.018
DM	6434 (7.09)	1699 (7.49)	0.015
CAD	14372 (15.8)	3700 (16.3)	0.013
Stroke	1911 (2.10)	497 (2.19)	0.006
Epilepsy	892 (0.98)	237 (1.04)	0.006
Head injury	6151 (6.78)	1629 (7.18)	0.016
RLS	113 (0.12)	27 (0.12)	0.002
Depression	7985 (8.80)	2041 (8.99)	0.007
Dementia	472 (0.52)	123 (0.54)	0.003
Asthma	7902 (8.70)	2051 (9.04)	0.012
COPD	10823 (11.9)	2781 (12.3)	0.010
**Medicine**			
Migraine prophylaxis	38321 (42.2)	16293 (71.8)	0.626

Migraine prophylaxis including amitriptyline, clomipramine, duloxetine, flunarizine, fluoxetine, gabapentin, imipramine, paroxetine, sertraline, topiramate, valproate, venlafaxine and beta blockers.

Abbreviation: DM: diabetes mellitus; CAD: coronary arterial disease; RLS: restless leg syndrome; COPD, chronic obstructive pulmonary disease

Table 2 shows the incidences and HRs of ET between the migraine and the migraine-free cohorts. A total of 152 and 319 ET events occurred in the migraine and the migraine-free cohorts respectively. The incidences of subsequent ET were 8.97 and 4.81 per 10000 person-years in the migraine and the migraine-free cohorts respectively.

**Table 2:** Incidence of subsequent essential tremor and multivariate Cox proportional hazards regression analysis measured hazard ratio for the study cohort.

Variable	Event	PYs	Rate	Crude HR (95% CI)	Adjusted HR (95% CI)
**Migraine**					
No	319	662554	4.81	ref	ref
Yes	152	169531	8.97	1.85 (1.53–2.24)	1.83 (1.50–2.23)
**Age group**					
20–44	120	480277	2.50	ref	ref
45–64	200	259087	7.72	3.09 (2.46–3.88)	2.49 (1.95–3.19)
≥65	151	92721	16.3	6.50 (5.11–8.26)	4.35 (3.21–5.90)
**Sex**					
Female	357	613662	5.82	ref	ref
Male	114	218423	5.22	0.90 (0.73–1.11)	0.83 (0.67–1.03)
**Hypertension**					
No	247	632119	3.91	ref	ref
Yes	224	199966	11.2	2.86 (2.39–3.43)	1.08 (0.85–1.36)
**DM**					
No	402	780050	5.15	ref	ref
Yes	69	52035	11.3	2.56 (1.98–3.30)	1.21 (0.92–1.58)
**CAD**					
No	308	711943	4.33	ref	ref
Yes	163	120142	12.4	3.12 (2.58–3.78)	1.32 (1.05–1.67)
**Stroke**					
No	457	819200	5.58	ref	ref
Yes	14	12885	10.9	1.92 (1.13–3.28)	0.58 (0.33–1.00)
**Epilepsy**					
No	455	825066	5.51	ref	ref
Yes	16	7018	22.8	4.10 (2.49–6.74)	3.06 (1.84–5.11)
**Head injury**					
No	427	781991	5.46	ref	ref
Yes	44	50094	8.78	1.59 (1.17–2.17)	1.30 (0.95–1.77)
**RLS**					
No	470	831351	5.65	ref	ref
Yes	1	734	13.6	2.39 (0.34–16.9)	1.32 (0.19–9.41)
**Depression**					
No	385	769697	5.00	ref	ref
Yes	86	62387	13.8	2.73 (2.16–3.46)	1.92 (1.51–2.45)
**Dementia**					
No	463	829336	5.58	ref	ref
Yes	8	2749	29.1	5.12 (2.55–10.3)	1.32 (0.64–2.72)
**Asthma**					
No	414	769389	5.38	ref	ref
Yes	57	62696	9.09	1.67 (1.27–2.21)	0.82 (0.61–1.11)
**COPD**					
No	353	745556	4.73	ref	ref
Yes	118	86528	13.6	2.86 (2.32–3.53)	1.53 (1.21–1.94)
**Migraine prophylaxis**					
No	142	407776	3.48	ref	ref
Yes	329	424309	7.75	2.24 (1.84–2.73)	1.10 (0.88–1.37)

Model adjusted for age (categorical), sex, hypertension, DM, CAD, stroke, epilepsy, head injury, RLS, depression, dementia, asthma, COPD and migraine prophylaxis

PYs: person-years; rate: incidence rate, per 10000 person-years; DM: diabetes mellitus; CAD: coronary arterial disease; RLS: restless leg syndrome; COPD, chronic obstructive pulmonary disease

**Fig 1:**
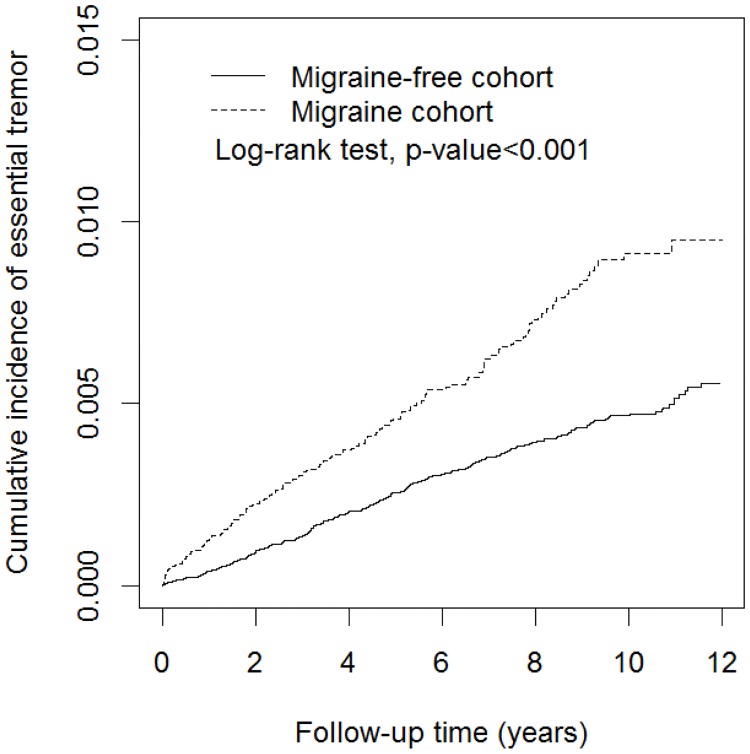
Cumulative incidence of subsequent essential tremor in the comparison and migraine cohorts.

Fig 1 demonstrates a significantly higher cumulative incidence for ET in the migraine cohort than the migraine-free cohort (log-rank test p < 0.0001). After adjusting for age, sex, comorbidities and migraine prophylaxis, the individuals with migraine were associated with a 1.83-fold higher risk of ET compared to the migraine-free individuals (HR = 1.83, 95% CI = 1.50–2.23). The risk of developing ET appears to increase with age. Individuals aged 45−64 and more than 65 had adjusted HRs of 2.49 (95% CI = 1.95–3.19) and 4.35 (95% CI = 3.21–5.90) compared with individuals aged 20−44. In additional, the results also demonstrated trends showing increasing risks of ET in subjects with CAD (HR = 1.32, 95% CI = 1.05–1.67), epilepsy (HR = 3.06, 95% CI = 1.84–5.11), depression (HR = 1.92, 95% CI = 1.51–2.45), and COPD (HR = 1.53, 95% CI = 1.21–1.94).

Table 3 shows the risks of ET in different tiers of frequency of annual medical visits due to migraine. After adjustment for age, sex, comorbidities and migraine prophylaxis, the HRs of subsequent ET were 1.57 (95% CI = 1.27–1.95), 5.42 (95% CI = 2.96–9.95) and 30.9 (95% CI = 18.3–52.2) for the migraineurs with < 6 times, 6–11 times and ≥ 12 times of annual visits respectively, in comparison to the individuals without migraine. Thus, there is a trend showing increasing risk of ET with increasing frequency of medical visits due to migraine (p for trend < 0.0001).

**Table 3:** Incidence of subsequent essential tremor and multivariate Cox proportional hazards regression analysis measured hazard ratio by various tiers of frequency of annual medical visits related to migraine.

Average annual medical visits related to migraine	N	Event	PYs	Rate	Adjusted HR (95% CI)
**Migraine-free cohort**	90784	319	662554	4.81	ref
**Migraine cohort**					
<6	21967	126	165489	7.61	1.57 (1.27–1.95)
6–11	531	11	3120	35.3	5.42 (2.96–9.95)
≥12	198	15	922	163	30.9 (18.3–52.2)
p for trend					<0.0001

Model adjusted for age (continuous), sex, hypertension, DM, CAD, stroke, epilepsy, head injury, RLS, depression, dementia, asthma, COPD and migraine prophylaxis

PYs: person-years; rate: incidence rate, per 10000 person-years

Table 4 demonstrates the risks of ET in the migraine cohort compared with the migraine-free cohort as stratified by age, sex, comorbidities and migraine prophylaxis. Comparing with the migraine-free cohort, the HRs of ET in migraineurs were 1.95 (95% CI = 1.32–2.89), 1.92 (95% CI = 1.43–2.58) and 1.53 (95% CI = 1.04–2.26) in the subgroups stratified by age 20–44 years, 45–64 years and ≥ 65 years respectively. Relative to the migraine-free cohort, the HRs of ET were 1.92 (95% CI = 1.52–2.41) and 1.71 (95% CI = 1.14–2.56) for female and male migraineurs respectively. Comparing with the migraine-free cohort, the HRs of ET were 2.08 (95% CI = 1.41–3.05) and 1.66 (95% CI = 1.31–2.09) for the migraine patients without any comorbidity and with at least one comorbidity respectively.

**Table 4:** Incidence of subsequent essential tremor and multivariate Cox proportional hazards regression analysis measured hazard ratio for the study cohort stratified by age, sex, and comorbidities.

	Migraine-free cohort			Migraine cohort			Adjusted HR
Variable	Event	PYs	Rate	Event	PYs	Rate	(95% CI)
**Age group**^a^							
20–44	76	385313	1.97	44	94964	4.63	1.95 (1.32–2.89)
45–64	127	201103	6.32	73	57985	12.6	1.92 (1.43–2.58)
≥65	116	76138	15.2	35	16582	21.1	1.53 (1.04–2.26)
**Sex**^b^							
Female	243	489931	4.96	114	123731	9.21	1.92 (1.52–2.41)
Male	76	172622	4.40	38	45801	8.30	1.71 (1.14–2.56)
**Comorbidity**^c^							
No	84	400716	2.10	44	88534	4.97	2.08 (1.41–3.05)
Yes	235	261838	8.98	108	80997	13.3	1.66 (1.31–2.09)

^a^Model adjusted for sex, hypertension, DM, CAD, stroke, epilepsy, head injury, RLS, depression, dementia, asthma, COPD, and migraine prophylaxis

^b^Model adjusted for age (continuous), hypertension, DM, CAD, stroke, epilepsy, head injury, RLS, depression, dementia, asthma, COPD and migraine prophylaxis

^c^Model adjusted for age (continuous), sex and migraine prophylaxis

PYs: person-years; rate: incidence rate, per 10000 person-years

## Discussion

In the present study, we found that patients with migraine were associated with a 1.83-fold increased risk of future ET, after adjusting for age, sex, comorbidities and migraine prophylaxis. In addition, the risk appeared to be highest in the youngest group, i.e. age between 20 to 44 years, with an adjusted HR of 1.93. More frequent migraine-related health-care-seeking behaviour was associated with higher incidence of ET.

Except for the study of Barbanti et al. [4], our finding is in accordance with the results of previous studies that support a link between migraine and ET (Table 5). As mentioned, the early cross-sectional studies were limited by the lack of control in sex and age [1, 2], whereas the latter case-control studies done in tertiary-referral centers may not reflect the condition in the general population [4, 5]. Notably, unlike the majority of previous studies (except Biary, Koller et al.[1]) that showed unidirectional association between both disorders by examining the lifetime prevalence of migraine in ET, the current study, however, investigated the occurrence of ET in migraine over the span of a decade. Comparing to previous studies, this is by far the largest and the only population-based study to demonstrate an association between migraine and ET.

**Table 5:** Summary of previous studies examining the association between migraine and ET.

Refence	Study	sex	N	Mean age (years)	Results
Biary et al. 1990	cross-sectional	Not controlled	1. ET group: ET vs. HC = 74 vs. 102	1. ET group:59	1. ET group: moremigraine in ET than HC (36% vs. 18%, *p* = 0.005)
			2. Migraine group: Migraine vs. HC = 58 vs. 85	2. Migraine group:39	2. Migraine group: more ET in migraine than HC (17.2% vs. 1.2%, *p* = 0.001)
			3. Control group: 108	3. Control group: migraine: 41; ET: 47	3. Control group: More ET in migraine vsnon-migraine (22% vs 1%, *p* = 0.002)
Bain et al. 1994	cross-sectional	Not controlled	73 ET (20index and 53 secondary cases from family)	54(index cases); 45(secondary cases)	26% (19)of ET had classical migraine
Duval et al. 2006	cross-sectional	M:F = 1:5	30migraine	41	No correlation between tremor characteristics and no. of years of migraine
Barbanti et al. 2010	Case- control	M:F = 1:3	ET: 110; HC: 110	68.3	similar lifetime or current migraine in both groups
Hu et al. 2014	Case- control	M:F = 1:1	ET: 150; HC: 150	50	Lifetime migraine higher in ET than HC (22% vs. 12.7%, p = 0.035; OR = 1.95)

HC: healthy control; M: male; F: female; no.: number

The mean age of our cohorts (at index date), i.e. 45 years, is younger than that of previous studies. Further analysis showed increased risks of ET among migraine cases across all age groups (i.e. <45, 45–64 and ≥65 years) compared to the migraine-free cohort. Notably, there seems to be an even stronger association, an adjusted HR of 1.95, in the youngest age group 20–44 years and a less prominent correlation in the oldest group (≥65 years), i.e. an adjusted HR of 1.53. The reason underlying this is obscure but could be due to the different age distribution of the two disorders.

Migraine is the most common neurological disorder, affecting approximately 15–20% of the general population [9, 10]. ET is estimated to have a prevalence of 0.3–4% in individuals ≥ 40 years old, with higher incidence of up to 14% in the elderly population [11]. ET was originally considered to be a “benign” monosymptomatic tremorogenic disorder (4-12Hz) with substantial hereditary basis [11]. Emerging evidence suggests that ET may have subtle neurological deficits beyond its hallmark of kinetic tremor [12]. These include cerebellar dysfunction, bradykinesia, disturbed ocular movements as well as non-motor features, namely voice disorders [13], upper airway dysfunction [14], impairment visual reaction time [15], mild cognitive deficits, impaired executive functions and neuropsychiatric symptoms [11, 12]. Neuroimaging studies have shown widespread alterations in the cerebello-thalamo-cortical circuit [16, 17], with cerebellar involvement as the most consistent finding [18]. Intriguingly, the cerebellum and brainstem circuits have also been implicated in the pathophysiology of migraine [19, 20]. Migraineurs, notably those with aura (MA), showed subclinical hypermetria and other cerebellar signs, suggestive of abnormal functioning of the lateral cerebellum [21]. Indeed, both MA and migraineurs without aura (MwA) showed increased prevalence of silent posterior circulation infarctions, with a majority of lesions located at the cerebellum [22]. The risk appeared to be strongest in cases with MA, female gender as well as higher attack frequency. As such, although the link between migraine and ET remains speculative, it might be possible that the increased risk of ET in migraine is mainly driven by MA, possibly via a shared pathophysiology that implicates the same cerebellar circuits. This notion was partially elucidated by a study showing no subtle alternation in physiological tremors of migraineurs compared to controls, regardless of the number of years experiencing migraine, suggesting that the possible link between migraine and ET might be the result of an acute event of the olivo-cerebellar circuit rather than a progressive alternation of tremorgenic mechanisms [3]. These speculations, however, should be interpreted with caution and require future pathological and imaging studies to provide definitive evidence.

Our findings demonstrated a staggeringly increased incidence of ET in migraineurs seeking more frequently for migraine-related health care. This cohort of subjects often consists of MA and chronic migraineurs who want to be reassured of the benign nature of their headaches as well as requesting for migraine prophylaxis. Both MA and chronic migraine are known to activate several extrapyramidal nuclei such as the red nucleus and substantia nigra during migraine attacks [23]. One plausible speculation is that repeated migraineurs attacks may lead to dysfunction or structural changes of these nuclei that may give rise to movement disorders. Intriguingly, a recent epidemiological study supported the notion that MA are more prone to develop Parkinson’s disease [24], a possible comorbidity of ET [25].

Arguably, our findings are based on a population mainly composed of the Han ethnic group and thus may not be applied to Caucasian due to the genetic differences, as demonstrated by the conflicting results between the Chinese [5] and Italian [4] studies. On the other hand, migraine and ET may share a common genetic background. For instance, a functional variant (Ser9Gly) of the dopamine D3 receptor (DRD3), although not confirmed in other studies[26], is associated with risk and age-at-onset of ET, the frequencies of the functional variant were found to be similar between ET patients with and without migraine [27]. Nevertheless, there is a scarcity of pathophysiological evidence to support the association between the two disorders. Future research should be undertaken to elucidate the potential common pathophysiology of migraine and ET.

### Strength of the current study

The major strength of the present study is that we analysed a large, national dataset containing a representative cohort of 1 000 000 citizens covered by the NHI of Taiwan. The large sample size and the long (10 years) observation period offered significant power for statistical analyses. In addition, we robustly controlled the confounding effects of migraine prophylaxis that would lead to misdiagnosis of ET because of their potential side effects of tremor. These commonly used medications included anticonvulsants (valproate acid, topiramate and gabapentin [28]), selective serotonin re-uptake inhibitors (SSRIs) (fluoxetine, sertraline, paroxetine), serotonin norepinephrine reuptake inhibitors (SNRI) (venlafaxine, duloxetine) (Morgan and Sethi 2005) [29] [30], tricyclic antidepressant (amitriptyline, clomipramine and imipramine) [31], calcium channel blocker (flunarizine) [32] and beta blockers [33]. To the best of our knowledge, this has not been done in previous studies.

### Limitations of the current study

First, we were unable to delineate between migraineurs with aura (MA) and migraineurs without aura because those two were not separately recorded in the NHIRD. As mentioned, the link between migraine and ET may be driven mainly by MA. Similarly, other factors such as family history, symptoms and severities of ET were impossible to be examined. Another concern is the accuracy of migraine and ET diagnoses. Nonetheless, the accuracy of the diagnoses might have been improved by the wide use of the ICHD-2 diagnostic criteria for migraine in Taiwan. The validity of the diagnoses was also improved by the routine verification of coding by the NHI Bureau of Taiwan for the eligibility of health insurance reimbursement. Third, another potential bias is that migraineurs with higher frequencies of seeking medical care for migraine would have had better chances in diagnosing with ET. Likewise, a longer follow-up might facilitate the diagnosis of ET in migraine patients under neurological surveillance. Last but not least, the NHIRD is used for health insurance claims and may not serve directly for scientific purposes. Future studies are warranted to replicate our findings.

## Conclusion

Our nationwide population-based cohort study showed that migraine increases the likelihood of incident ET. This provides additional evidence to support the link between migraine and may suggest the presence of shared pathophysiology, whereby increasing the vulnerability of developing one condition with another. Yet much remains unexplained, future genetic, pathological imaging and epidemiological studies are warranted to explore the underpinning networks that potentially link these two common but complex neurological disorders.

## Supporting information

S1 STROBE Checklist Checklist of items that should be included in reports of observational studies. (DOC)

## Abbreviations

CI: confidence interval
ET: essential tremor
HR: hazard ratio
ICD-9-CM: International Classification of Diseases, Ninth Revision, Clinical Modification
NHIRD: National Health Insurance Research Database
